# A graphene oxide/amidoxime hydrogel for enhanced uranium capture

**DOI:** 10.1038/srep19367

**Published:** 2016-01-13

**Authors:** Feihong Wang, Hongpeng Li, Qi Liu, Zhanshuang Li, Rumin Li, Hongsen Zhang, Lianhe Liu, G. A. Emelchenko, Jun Wang

**Affiliations:** 1 Key Laboratory of Superlight Material and Surface Technology, Ministry of Education, Harbin Engineering University, 150001, P. R. China; 2 Institute of Advanced Marine Materials, Harbin Engineering University, 150001, P. R. China; 3 Institute of Solid State Physics, Russian Academy of Sciences, Chernogolovka 142432, Russia

## Abstract

The efficient development of selective materials for the recovery of uranium from nuclear waste and seawater is necessary for their potential application in nuclear fuel and the mitigation of nuclear pollution. In this work, a graphene oxide/amidoxime hydrogel (AGH) exhibits a promising adsorption performance for uranium from various aqueous solutions, including simulated seawater. We show high adsorption capacities (Q_m_ = 398.4 mg g^−1^) and high % removals at ppm or ppb levels in aqueous solutions for uranium species. In the presence of high concentrations of competitive ions such as Mg^2+^, Ca^2+^, Ba^2+^ and Sr^2+^, AGH displays an enhanced selectivity for uranium. For low uranium concentrations in simulated seawater, AGH binds uranium efficiently and selectively. The results presented here reveal that the AGH is a potential adsorbent for remediating nuclear industrial effluent and adsorbing uranium from seawater.

Uranium, a significant strategic and energy resource, has attracted increasing attention. Nuclear power is considered a feasible solution to the global energy problem caused by the exhaustion of fossil fuel. Global warming is another main driving force that has prompted many countries to develop their nuclear industries to address energy security and reduce greenhouse gas emissions^1^. The recovery of uranium (VI) from alternative uranium reserves, such as seawater, underground water and nuclear waste solutions, has become imperative due to the limited supply of uranium from terrestrial ores and the minimal environmental cost^2^,^3^,^4^. Among the alternative uranium reserves, the largest source of uranium is seawater, as the concentration of soluble uranyl ions in seawater is extremely high, 3.3 μg L^−1 ^5,6. Meanwhile, excessive amounts of uranium (VI) have been inevitably released into the environment through activities of the nuclear industry, threatening human health and the survival of bio-organisms^7^. Nuclear leakage in Fukushima raised heated concern for the safe disposal of radioactive substances to protect the environment^8^. For years, various techniques such as precipitation, membrane dialysis, solvent extraction, ion exchange and adsorption have been developed to minimize uranium (VI) pollution from aqueous solutions^9^,^10^,^11^. Among these methods, adsorption appears to be a cost-effective approach for removing heavy metal ions as a part of wastewater management.

Different types of adsorbents have been developed for uranium (VI) removal in an economical manner, such as inorganic oxides^12^,^13^, metal-organic frameworks^14^,^15^ and biomass^16^,^17^. However, most materials in groups (i), (ii) and (vi) have no resistance to the acidic environment, and those in group (iii) have no resistance to radiation^18^. For these reasons, carbon materials are promising candidates for use because of their thermal and radiation stabilities^19^. Graphene, a monolayer of graphite, with its advantageous thermal and mechanical properties, has been used in many areas, such as electrochemical energy storage, solar cells and gas adsorption^20^,^21^,^22^. As a result of its lack of targeted functional groups, the maximum adsorption capacity of graphene oxide (GO) for uranium (VI) is only 97.5 mg g^−1 ^23,24.

Amidoxime, one of the most effective chelating groups, has attracted increasing attention for application in uranium (VI) removal. Membranes, macroporous fibres and composites containing amidoxime groups have been prepared by radiation-induced grafting, suspension and sonochemical functionalization^25^,^26^,^27^. According to recent studies, amino acids, polyamines and metformin containing multiple nitrogen basic moieties bind with the –COOH or –OH groups onto GO sheets by hydrogen binding or acid-base-type electrostatic attraction, forming an assembly of GO nanosheets into hydrogels^28^,^29^,^30^. In this work, we prepared a graphene oxide/amidoxime composite hydrogel (AGH) by directly mixing the dispersions of both components and explored its application as an adsorbent for uranium (VI). In addition, the affinity and selectivity for UO_2_^2+^ were tested in a solution containing other cations and simulated seawater. Based on the experimental results, AGH is a potential adsorbent for remediating nuclear industrial effluent and adsorbing uranium from seawater.

## Results and Discussion

### Morphology and characterization

**Figure 1:**
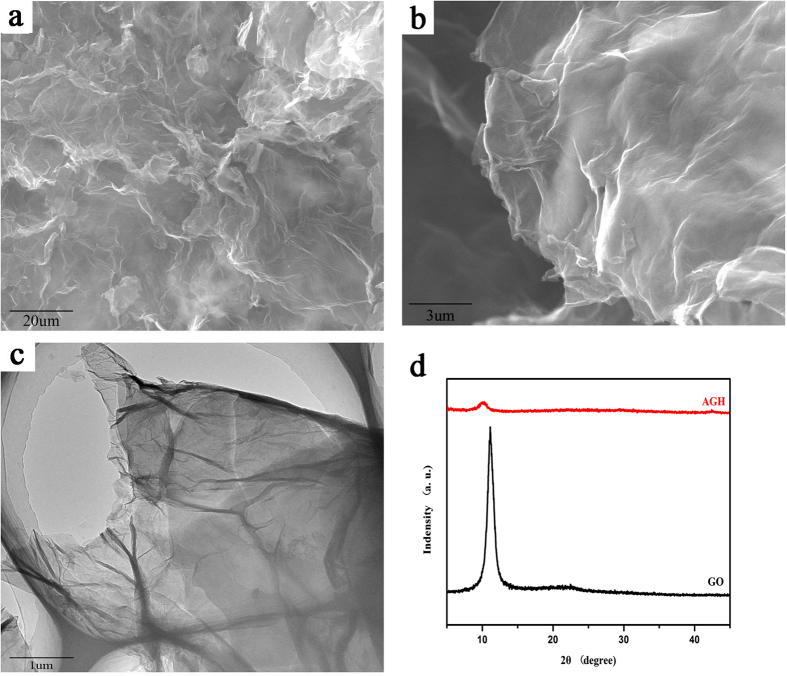
SEM images of (a,b) AGH, (c) TEM image of AGH, (d) XRD patterns of GO and AGH.

A novel gel with AO–IDAN as a crosslinking agent can be fabricated by introducing an appropriate amount of its dispersion into a GO dispersion (Supplementary Information S1). It can be seen that AO–IDAN can promote the self-assembly of GO into a hydrogel. The amino groups (–NH_2_) of AO-IDAN may accept protons from the carboxylic acid groups (–COOH) of the GO sheets to participate in acid–base-type electrostatic attractions. More importantly, the amino groups (–NH_2_) and hydroxyl groups (–OH) of AO-IDAN can also form strong hydrogen bonds with the hydroxyl groups (–OH) and carboxylic acid groups (–COOH) of the GO sheets^31^,^32^. The morphology of AGH was investigated by scanning electron microscopy (SEM) and transmission electron microscopy (TEM). As shown in Fig. 1a,b, AGH exhibits a porous structure of overlapping GO nanosheets; observations by TEM confirm these findings (Fig. 1c). As shown in Fig. 1d, XRD patterns indicate an apparent difference between GO and AGH. GO has a sharp and strong characteristic X-ray diffraction peak of approximately 2θ = 11.4°, typical for a layered structure of GO nanosheets. For AGH, by comparison, the peak becomes less pronounced and shifts to approximately 2θ = 9.62° because of the random organization of GO nanosheets into a 3D network^33^. The specific surface areas and the pore size distribution plots of AGH were obtained using the Brunauer-Emmett-Teller (BET) method *via* N_2_ adsorption isotherms and the Barrett-Joyner-Halenda (BJH) method (Supplementary Information S2). The shape of the isotherm is classified as type IV, indicating the porous structure of AGH. The surface area and pore volume of AGH are 58.69 m^2^/g and 0.068546 cm^3^/g, respectively. The pore size distribution curves of AGH also show the size distribution of the AGH.

**Figure 2:**
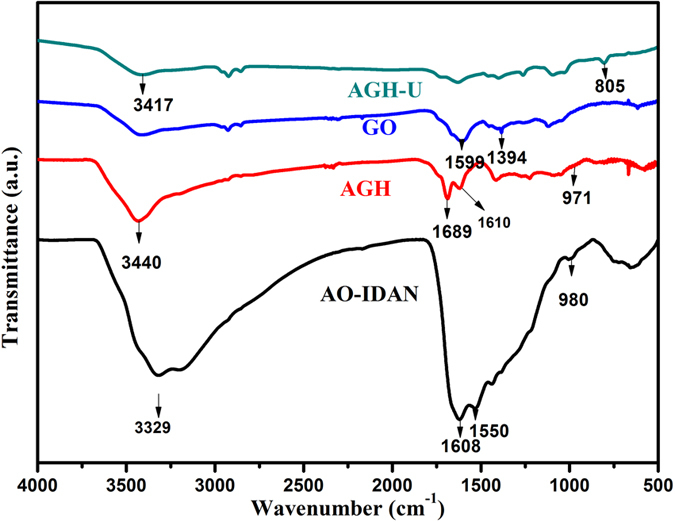
FT-IR spectra of AO-IDAN, GO AGH and AGH after adsorption of uranium.

**Figure 3:**
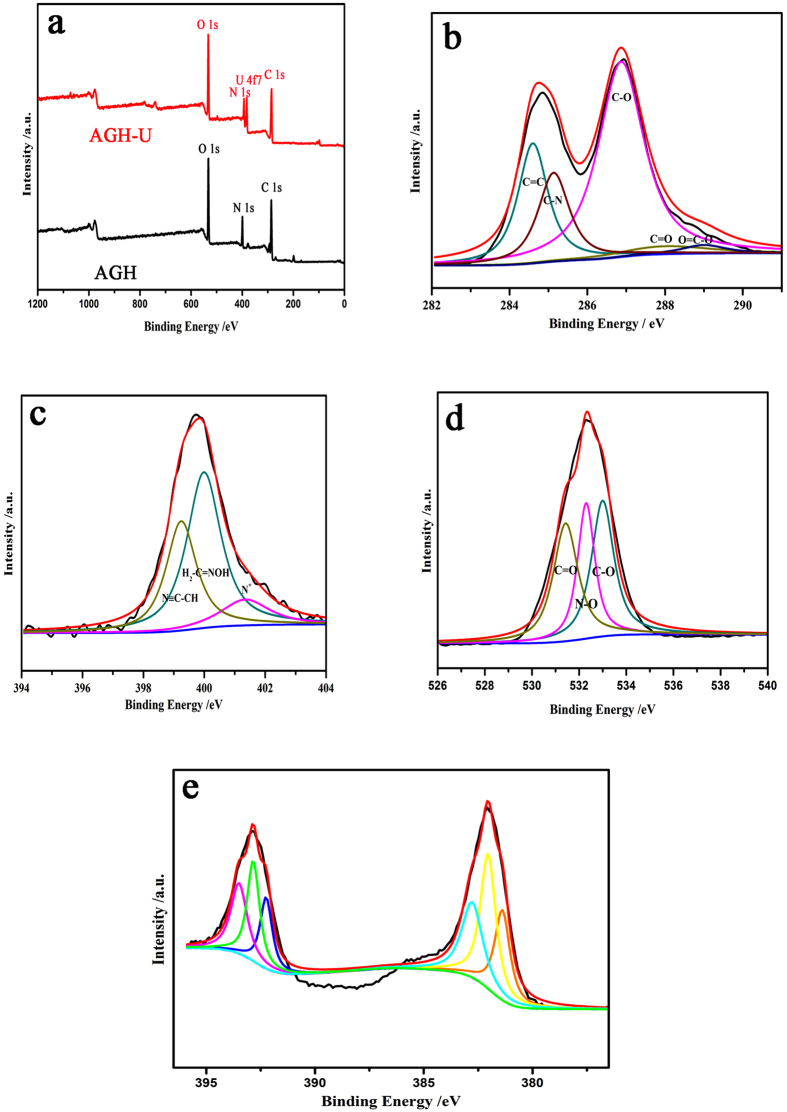
XPS spectra of (a) AGH and AGH after uranium adsorption; (b–d) high-resolution XPS spectra of C1s, N1s and O1s; (e) U4f_7/2_ and U4f_5/2_ spectra.

Fourier transform infrared spectroscopy (FT-IR) was used to identify the surface functional groups of GO, AO-IDAN, AGH and AGH after uranium adsorption (Fig. 2). The peaks at 1599 and 1394 cm^−1^ are attributed to the characteristic bands of GO^34^. The two bands at 1608 and 980 cm^−1^ that appear in AO-IDAN correspond to the C=N and N−O stretching vibrations of the amidoxime groups, which shift to 1610 and 971 cm^−1^, respectively. The vibrations of –OH or –NH_2_ at 3440 cm^−1^ and –C=O at 1689 cm^−1^ are also found in AGH. Due to the adsorption of uranium, some band shifts were observed. Meanwhile, there is a new peak at 805 cm^−1^ on the FTIR spectrum for AGH-U, which may be attributed to the antisymmetric stretching vibration of O=U=O^35^ and is significantly red-shifted compared with the corresponding peak for the aqueous UO_2_^2+^ complexes^6^. To verify the above FT-IR analysis, X-ray photoelectron spectroscopy (XPS) was used to investigate the chemical composition. The XPS survey spectra in Fig. 3a illustrate the coexistence of C, N, O and U. The high-resolution C1s spectrum (Fig. 3b) can be deconstructed into four individual peaks at 284.6, 285.2, 286.4 and 288.0 eV, assigned to C−C, C-N, C−O and C=O, respectively^36^. In a similar manner, the N1s spectrum (Fig. 3c) can be fitted to three typical peaks at 398.2, 399.4 and 400.2 eV, assigned to C=N, N≡C−CH and H_2_N−C=NOH, respectively^37^. There are also three fitted peaks in the O1s spectrum (Fig. 3d) centred at 531.5, 532.4 and 533.0 eV, which represent the C=O, N−O and C−O bands^38^. The presence of uranyl ions was further demonstrated by XPS, which reveals the presence of U4f_7/2_ and U4f_5/2_ peaks corresponding to binding energies of 382.0 eV and 392.8 eV (Fig. 3e). These binding energies are consistent with U^6+ ^39.

### Effects of pH on adsorption

**Figure 4:**
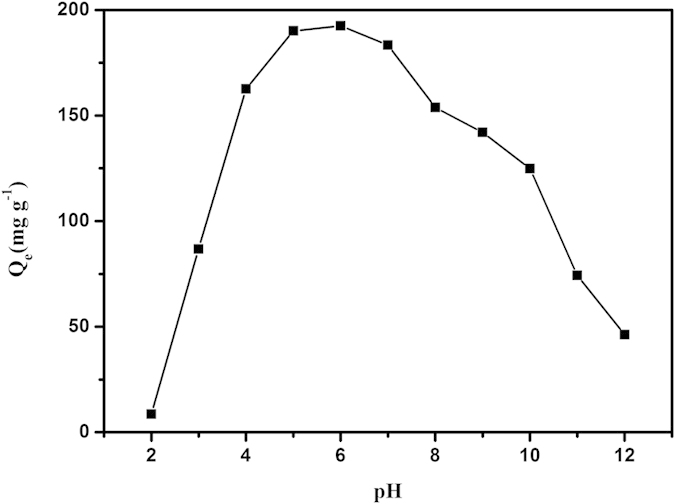
Effect of pH on the adsorption of uranium (VI) by AGH. Temperature 25 °C; amount of AGH 0.01 g; initial uranium concentration 100 mg L^−1^; volume of solution 20 mL.

pH has a substantial impact on the uranium (VI) adsorption process. pH affects the speciation of uranium in aqueous solution as well as the binding sites on the surface of the adsorbent^40^. The effect of pH on the adsorption of uranium (VI) by AGH was determined for the pH range of 2.00–12.00. The results show a strong influence of pH on adsorption. The amount of adsorbed uranium increases sharply from pH 2.00 to 5.00 and then remains relatively constant at pH 5.00–7.00 and decreases slowly with further increases in pH (Fig. 4). At pH ≤ 4.00, UO_2_^2+^ is the predominant form of uranium (VI) in solution. Taking the competition of H^+^ ions into account, uranium has few binding sites on the adsorbent surfaces^41^. As the pH increases, the degree of protonation of the oxime group most likely weakens, and the hydroxyl proton in the oxime group easily strips off to allow the lone pair of electrons on the negatively charged oxygen to occupy the empty orbitals of the uranium atom, thereby increasing the adsorption capacity^42^. When the pH value exceeds 8.00, the adsorbed amount falls off. The uranium (VI) anions such as UO_2_(OH)_3_^−^ and UO_3_(OH)_7_^−^ increase, leading to an electrostatic repulsion between these anions and the negatively charged surfaces of AGH at high pH. As a consequence, the optimum pH of the solution is 6.00 for the adsorption of uranium (VI) on AGH.

### Adsorption kinetics

**Figure 5:**
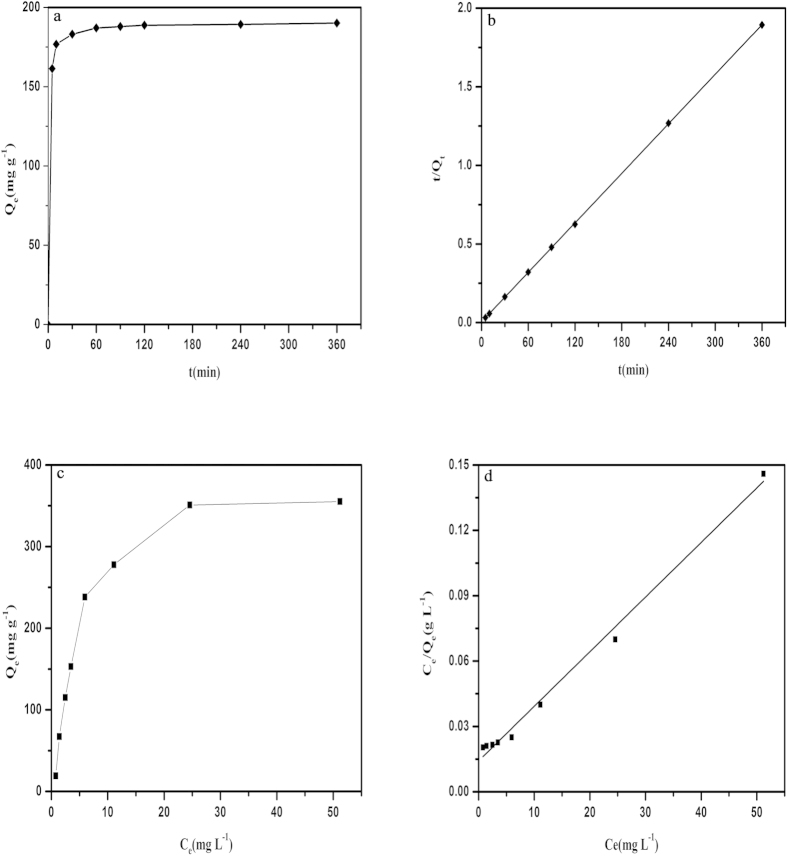
(**a**) Effect of contact time on uranium (VI) adsorption. (**b**) pseudo-second-order model for the adsorption of uranium (VI) on AGH. (**c**) Adsorption isotherm of AGH for uranium (VI). (**d**) Langmuir model for the adsorption of uranium (VI) by AGH.

Pseudo-first-order and pseudo-second-order models were used to examine the experimental data to determine the mechanism of the adsorption process (Supplementary Information S3). These results (Fig. 5a,b) show that the adsorption kinetics obeys a pseudo-second-order model with reasonable linearity, demonstrating the likelihood of chemical adsorption. Clearly, we see that AGH adsorbs more than 90% of the uranium (VI) in 60 min at V/m = 2000 mL/g.

### Adsorption isotherm

Adsorption isotherm models have been widely used to determine the relationship between the initial uranium (VI) concentration (C_o_) and equilibrium sorption capacity (Q_e_). The equilibrium data were analysed according to the Langmuir and Freundlich models (Supplementary Information S4). These results (Fig. 5c,d) show that the Langmuir isotherm is more applicable in characterizing the adsorption of uranium (VI) by AGH according to the R^2^ (0.99). The maximum adsorption of AGH synthesized here is calculated to be 398.41 mg g^−1^ and is larger than that of some adsorbents containing amidoxime groups^43^,^44^,^45^.

### Uranium adsorption from simulated seawater

Many cations, such as Mg^2+^, Ca^2+^, Ba^2+^ and Sr^2+^, exist in high concentrations in wastewater and may strongly compete with uranium (VI) for the selectivity of sorbents. Consequently, we investigated the effects of these cations on adsorption with MgCl_2_, CaCl_2_, Ba(NO_3_)_2_ and SrCl_2_ salts. We find that the distribution coefficient K_d_ used for the determination of the affinity and selectivity of AGH for uranium (VI) is greater than 1.89 × 10^5^ and compares well with those of the best uranium sorbents (Supplementary Information S5)^46^,^47^. This is because that amidoxime is an excellent chelating functional group due to the presence of both acidic oxime and basic amino groups. The UO_2_^2+^ cation has a linear shape and favours co-ordination with ligands in the plane orthogonal to the O=U=O axis. It is proposed that the lone pairs of electrons of the amino nitrogen atom and the oxime oxygen atom can be donated to the positive metal centre to form a stable chelate (Supplementary Information S6)^48^. Certainly, the adsorption of uranium by GO also contributes to the uranium adsorption^23^.

Because of the excellent uranium (VI) adsorption properties described above, we sought to investigate the applicability of AGH for capturing trace amounts of uranium from seawater at trace concentrations of uranyl ions, as uranyl ions exist in the form of tricarbonate complexes [UO_2_(CO_3_)_3_^4−^]^49^. Selected results are shown in Table 1. Initially, we examined the performance of AGH for simulated seawater with sea salts (volume (V) of solution to mass (m) of AGH (V: m) = 1000 mL/g) to which ppb levels of uranium (~50 ppb) were added. From our studies, the quantitative removal of such low uranium levels is greater than 98%. Finally, we tested the ability of AGH to adsorb uranium at the low levels of naturally occurring uranium (~3.71 ppb for the test samples). The results indicate a remarkable efficiency of AGH to adsorb uranium at this extremely low level (removal capacities ~ 83% at V:m = 2000 mL/g, ~98% at V:m = 1000 mL/g ). This is encouraging for the use of AGH to capture uranium from seawater^50^.

In summary, a novel graphene oxide/amidoxime hydrogel was successfully synthesized by directly mixing dispersions of both components, with its structure well characterized. Compared to GO, AGH exhibits a higher adsorption capacity of uranium (VI) from aqueous solution. The maximum sorption capacity was evaluated to be 398.41 mg g^−1^ at pH 6.00 according to Langmuir isotherms. The adsorption process was well fitted with pseudo-second-order kinetics. In simulated seawater with different concentrations of uranium (VI) at ppb levels, AGH exhibited a high affinity and selectivity for uranium (VI), showing the potential for capturing uranium from seawater. In view of its excellent adsorption properties, AGH is a promising adsorbent practical application in the adsorption of uranium (VI) from aqueous solutions.

## Methods

### Preparation of AGH

All chemical agents in use were of analytical grade and without purification. GO was prepared from spectral graphite using the Hummers method^51^. NH_2_OH solution was prepared by dissolving 2.78 g NH_2_OH·HCl into a mixed solution of 50/50 (V/V)% water/ methanol and adjusting the pH to 7.0 by K_2_CO_3_. Then, 1.92 g iminodiacetonitrile (IDAN) was added into the solution, and the mixture was stirred at 80 °C for 8 h to generate an amidoxime dispersion (AO-IDAN). To prepare GO/amidoxime hydrogels, 4 mL GO solution (8 mg mL^−1^) was mixed with 2 mL of the amidoxime solution mentioned above, and the blended solution was shaken for approximately 10 s to form a hydrogel. The as-obtained AGH was freeze-dried for 4 days and dipped into distilled water and ethanol for 1 day, and then dried in a vacuum oven at 60 °C for 12 h.

### Characterization methods

The morphology and microstructure of AGH were investigated by scanning electron microscopy (SEM, JEOL JSM-6480A) and transmission electron microscopy (FEITEM, Tecnai G^2^ 20S-Twin). The samples were characterized by X-ray diffraction (XRD, Rigaku TTR-III) with high-intensity Cu-Kα radiation, operated at 40 kV and 150 mA in the range of 5–90° (2θ) at a scanning rate of 10 min^−1^. An ANATAR 360 FT-IR spectrophotometer was used to record the Fourier-transform (FT-IR) spectra by a standard KBr pellet technique. X-ray photoelectron spectroscopy (XPS) measurements were performed by a Thermo ESCALAB 250Xi spectrometer with monochromated Al Kα radiation (hv = 1846.6 eV). The binding energy scale of the spectrometer was calibrated using the metallic Cu 2p3/2 lines and Ag Fermi Edge of the respective reference metals. The binding energy of C1s (284.8 eV) was used as a reference to eliminate charge effects. A Bruker-MS ICP-MS instrument was used to determine the concentration of uranium (VI) in aqueous solutions.

### Adsorption experiments

To investigate the influences of the conditions of the uranium (VI) adsorption, the pH, the contact time and initial ion concentration were studied in a bath system. AGH (0.01 g) was added to a conical flask containing 20 mL uranium (VI) solution at a given pH value and concentration. The pH was adjusted by 0.1 mol/L NaOH or HNO_3_. In addition, simulated sea water was prepared by dissolving 33.3 g sea salts into 1 L distilled water, and the concentration of uranyl ions was adjusted by uranyl nitrate. The flask was shaken at room temperature for 6 h in a thermostatic shaker bath. The suspension was centrifuged at 6000 rpm for 5 min. The concentration of uranium (VI) in the suspension was obtained by a Bruker 820-MS ICP-MS instrument. The adsorption amount of uranium (VI) Q_e_ (mg g^−1^) and the % removal were calculated according to eqs (1) and (2):


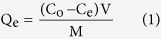



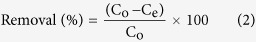


in which C_o_ (mg L^−1^) represents the uranium (VI) ion concentration in the initial solution, C_e_ (mg L^−1^) represents the uranium (VI) ion concentration at equilibrium, V (L) is the volume of the solution in use and M stands for the weight of adsorbent (g).

## Additional Information

**How to cite this article**: Wang, F. *et al.* A graphene oxide/amidoxime hydrogel for enhanced uranium capture. *Sci. Rep.* **6**, 19367; doi: 10.1038/srep19367 (2016).

## Supplementary Material

Supplementary Information

## Figures and Tables

**Table 1:** Selected results for the extraction of uranyl ions from simulated seawater.

V:m (mL/g)	U concentration (ppb)		%Removal
	initial	final	
1000	102.77	1.57	98.47
1000	57.28	0.52	99.10
1000	3.71	0.05	98.65
2000	57.28	1.93	96.63
2000	3.71	0.62	83.29
