# Supplementary material for: A graphene oxide/amidoxime hydrogel for enhanced uranium capture
Source: Sci Rep. 2016 Jan 13;6:19367. doi: 10.1038/srep19367 (PMC4725816; doi:10.1038/srep19367)

**Supplementary Information for**

A graphene oxide/amidoxime hydrogel for enhanced uranium capture

Feihong Wang,*^a^* Hongpeng Li, *^a^* Qi Liu, *^a^* Zhanshuang Li,**^a^* Rumin Li, *^a^* Hongsen Zhang, *^a^* Lianhe Liu*, ^b^* G.A. Emelchenko*^c^* & Jun Wang**^a,b^*

^a^Key Laboratory of Superlight Material and Surface Technology, Ministry of Education, Harbin Engineering University, 150001, P. R. China, ^b^Institute of Advanced Marine Materials, Harbin Engineering University, 150001, P. R. China, ^c^Institute of Solid State Physics, Russian Academy of Sciences, Chernogolovka 142432, Russia

**S1 Photographs of graphene oxide/amidoxime mixtures with different volume ratio of amidoxime to Go, from left to right, 0:4, 0.5:2, 2:4, 2.5:4.**


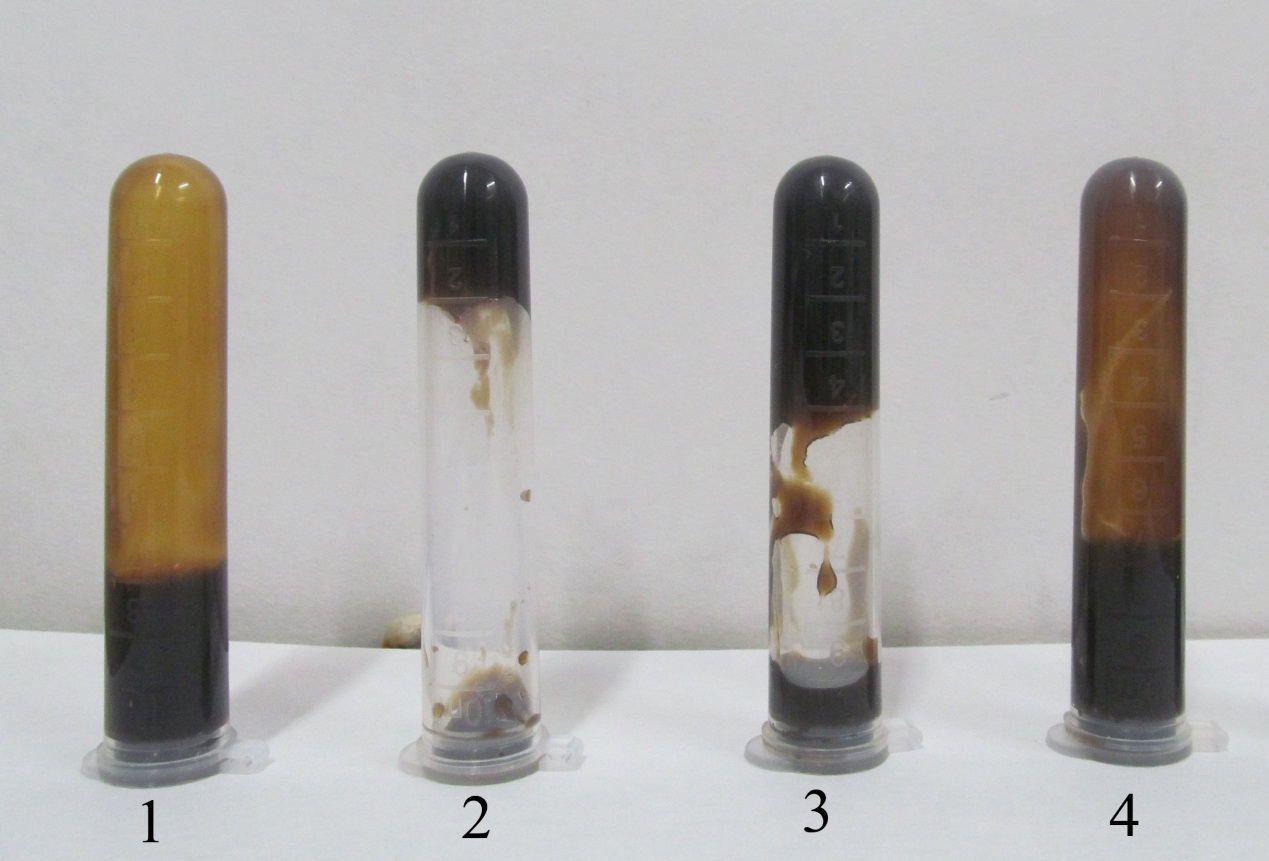


**S2 The N_2_ adsorption/desorption isotherms (a) and pore size distributions of AGH (b).**


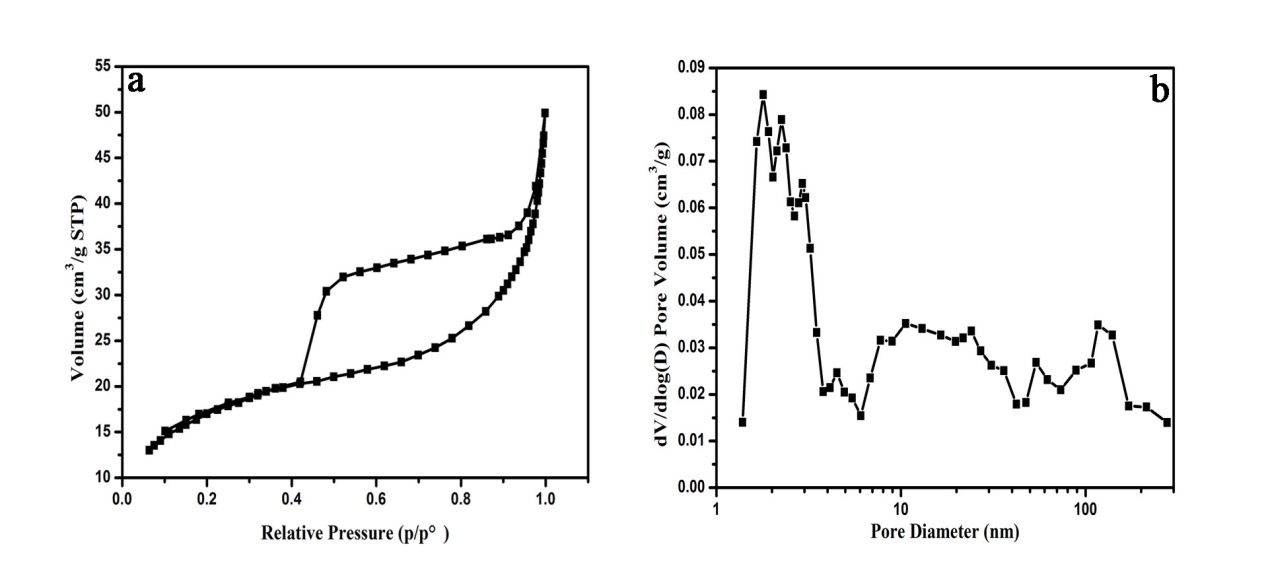


**S3 Adsorption kinetics**

The pseudo first-order model is usually applied to the initial stage of an adsorption process. The model is expressed by the following equation

(1)

where and are the amounts of uranium adsorbed (mg/g) at equilibrium and at any time, $t$ (min), respectively, and is the equilibrium rate constant of the pseudo first-order sorption (1/min).

The equation of pseudo second-order model is illustrated as

(2)

where (g/mg/min) is the equilibrium rate constant of pseudo second-order. (mg/g) is the maximum adsorption capacity and (mg/g) is the adsorbing capacity at time $t$ (min).

**Determined Constants of Pseudo-first-order and Pseudo-second-order kinetic Models of Uranium adsorption onto AGH for 100 mg/L initial concentration.**

| **Pseudo-first kinetics model** | | | **Pseudo-second kinetics model** | | |  |
| --- | --- | --- | --- | --- | --- | --- |
| k_1_  (min^-1^) | Q_e_  mg/g) | R^2^ | k_2_  (g/mg·min) | Q_e_  (mg/g) | R^2^ | Q_e_-exp  (mg/g) |
| 0.01387 | 12.5484 | 0.754 | 0.00591 | 190.476 | 0.9999 | 190.092 |

**Pseudo first-order Model (a) and Pseudo second-order Model (b) for AGH.**


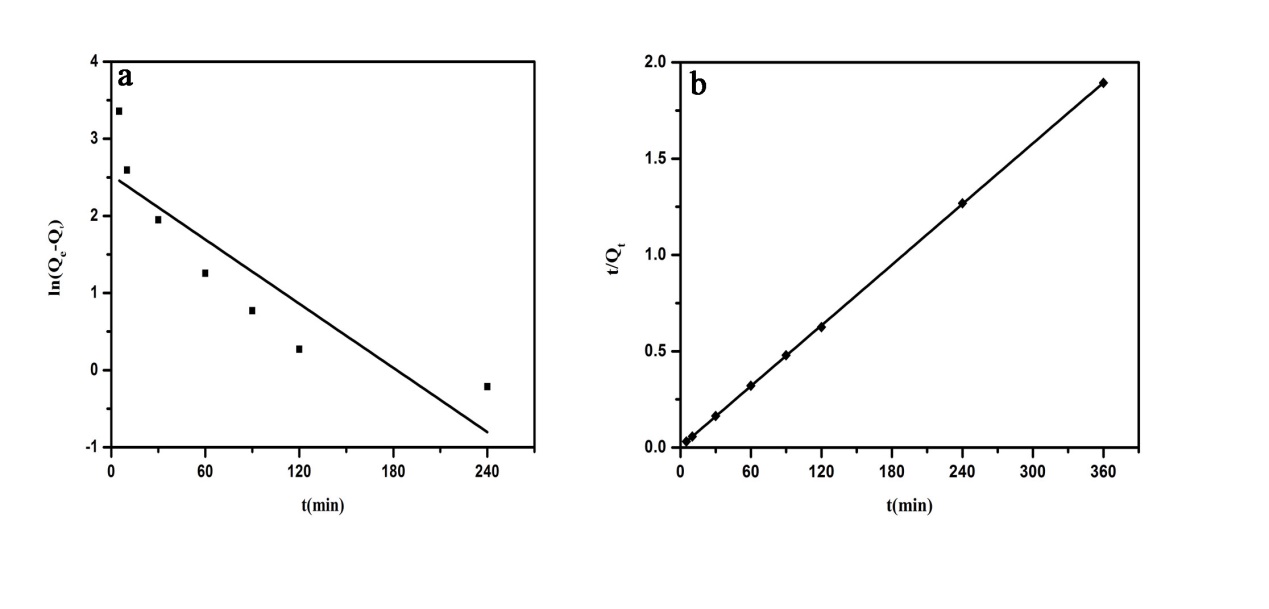


**References:**

1. Ho, Y.S., McKay, G. The kinetics of sorption of divalent metal ions onto sphagnum moss peat. *Water. RES.* **34**, 735-742 (2000).

2. Tan, L.CH., Wang, Y.L., Liu, Q., Wang, J., Jing, X.Y., Liu, L.H., Liu, J.Y. & D. Song, Enhanced adsorption of uranium (VI) using a three-dimensional layered double hydroxide/graphene hybrid material. *Chem. Eng. J.* **259**, 752-760 (2015).

**S4 Adsorption isotherm model**

The Langmuir mode predicts that each active site can only hold one adsorbate molecule and that the adsorption takes place on homogeneous sites within the adsorbent. There is no interacting between the adsorbed species. The linear equation given by the Langmuir is expressed as

(3)

where (mg/g) and (mg/L) are the capacity of adsorbed uranium onto samples and uranium concentration at equilibrium, respectively. (mg/g) is the maximum capacity of uranium adsorbed per unit mass of samples, and $b$(L/mg) is a constant related to the adsorption energy. Plotting versus generates a straight line with the slope and intercept , then and$b$are obtained.

The Freundlich mode is based on the assumption that the adsorption takes place on heterogeneous surface which have different adsorption energies. This model is expressed by

(4)

where is a constant related to adsorption capacity (mg/g) and $1/n$ is an empirical parameter connected to surface heterogeneity or adsorption intensity. It is generally stated that values of $n$ in the rang 2-10 means good, 1-2 moderately difficult, and less than 1 poor adsorption characteristics^1^. Additionally, the Freundlich mode in linear form is

(5)

the Freundlich constants and $1/n$ can be determined from the intercept and slope of linear plot of against l, respectively.

**Langmuir and Freundlich Isotherm Prameters for Uranium (VI) onto AGH.**

| **Sample** | **Langmuir** | | | **Freundlich** | | |
| --- | --- | --- | --- | --- | --- | --- |
|  | **Q_max_ (mg/g)** | **B**  **(L/mg)** | **R^2^** | **K_F_**  **(mg/g(L/mg)^1/n^)** | **n** | **R^2^** |
| AGH | 398.41 | 0.177 | 0.992 | 46.985 | 1.544 | 0.788 |

Langmuir (a) and Freundlich (b) models for the adsorption of uranium (VI).


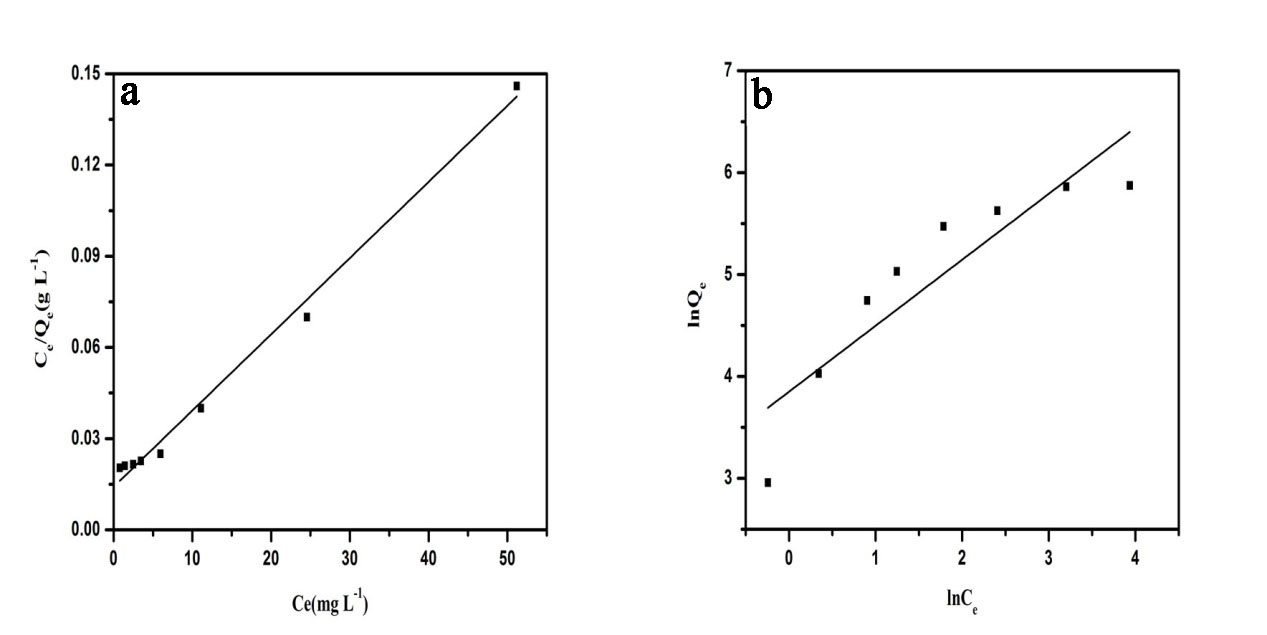


**References:**

1. Langmuir, I. The adsorption of gases on plane surfaces of glass, mice and platinum. *J. Am. Chem. Soc.* **40**, 1361-1403 (1918).

2. Yan, H.J., Bai, J.W., Chen, X., Wang, J., Zhang, H.S., Liu, Q., Zhang, M.L. & Liu, L.H*.* High U(VI) adsorption capacity by mesoporous Mg(OH)2 deriving from MgO hydrolysis. *RSC Adv.* **3**, 23278-23289 (2013).

3. Tan, L.CH., Liu, Q., Song, D.L., Jing, X.Y., Liu, J.Y., Li, R.M., Hu, S.X., Liu, L.H. & Wang, J. Uranium extraction using a magnetic CoFe2O4–graphene nanocomposite: kinetics and thermodynamics studies. *New. J. Chem.* **39**, 2832-2838 (2015).

**S5 Distribution coefficient.**

The distribution coefficient K_d_, used for the determination of the affinity and selectivity of AGH for uranium (VI), is given as

(6)

where C_o_ and C_e_ are the initial and equilibrium concentration of Mn^+^ (ppm), V is the volume (mL) of the testing solution, and m is the amount of the adsorbent (g) used in the experiment.


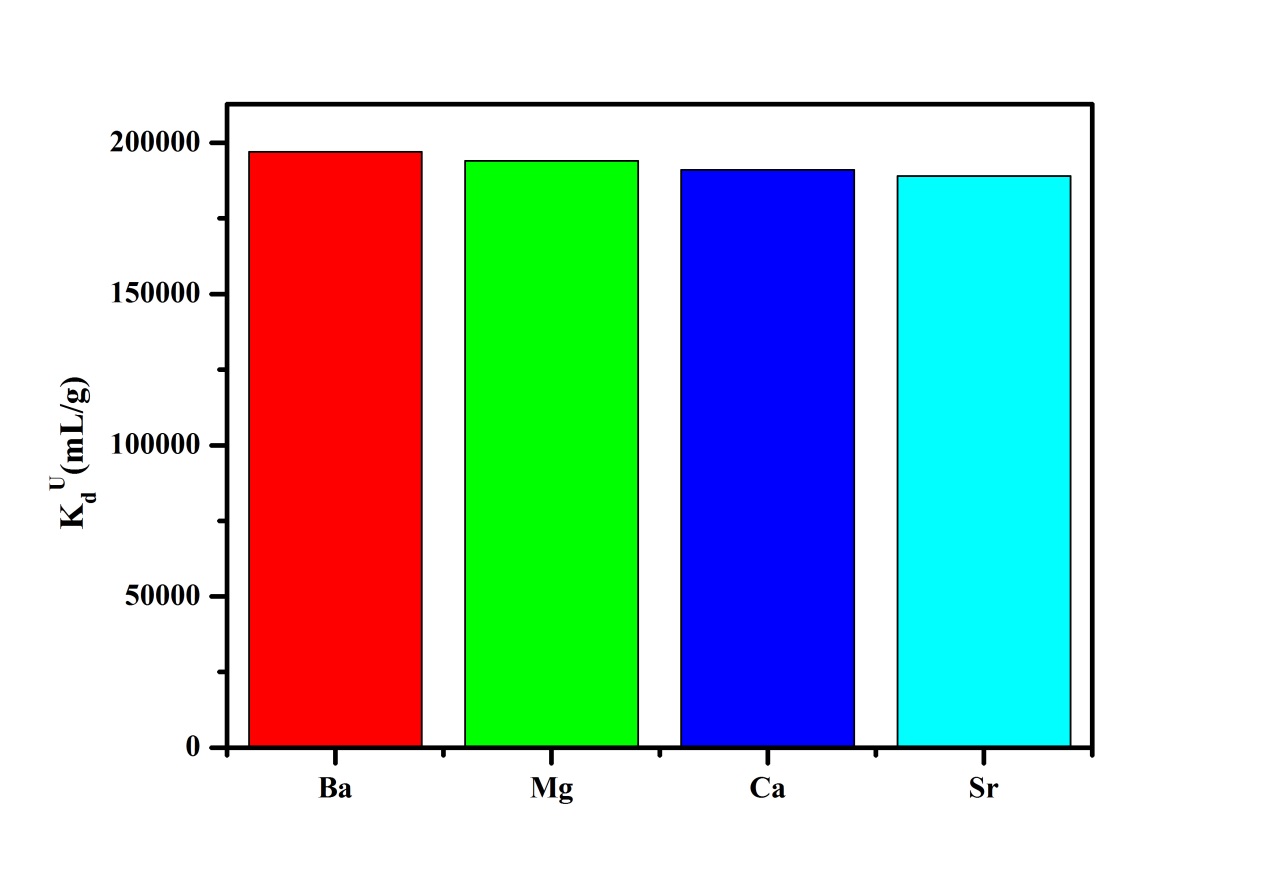
**The variation of the distribution coefficient K_d_^U^ (mL/g). (C_o_=5000mg/L for Ba^2+^, Mg^2+^, Ca^2+^ and Sr^2+^, C_o_=100mg/L for uranium; pH 6.0, m 0.01g; )**

**References:**

1 Al-Attar, L. & Dyer, A. Sorption behaviour of uranium on birnessite, a layered manganese oxide. *J. Mater. Chem.* **5**, 1381-1386 (2002).

2 Manos, M. J. & Kanatzidis, M. G. Layered metal sulfides capture uranium from seawater. *Journal of the American Chemical Society* **134**, 16441-16446 (2012).

**S6 Schematic description.**


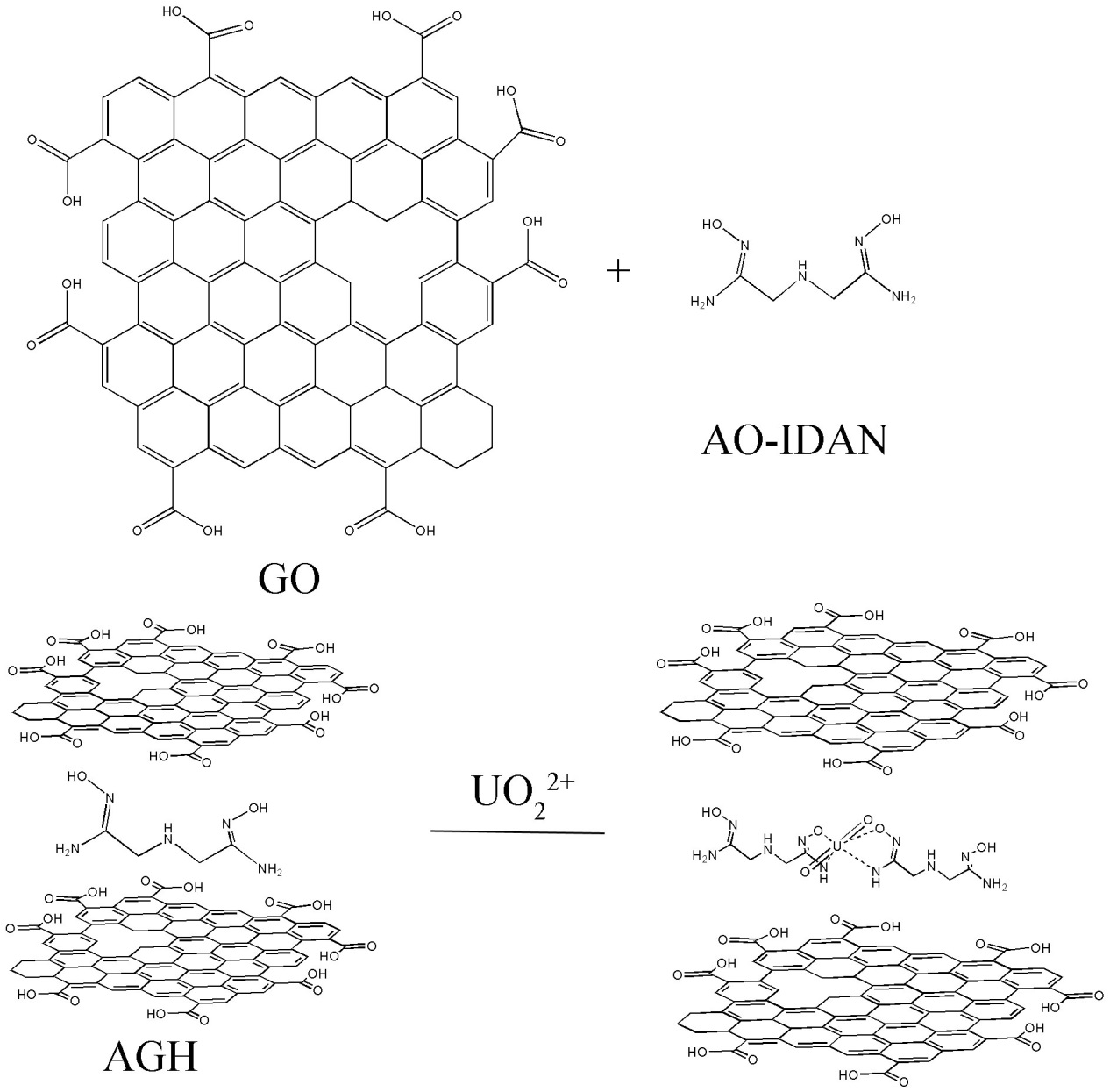

Supplement: Supplementary Information [file srep19367-s1.doc]
